# Optimization of Automated Radiotherapy Planning for Head and Neck Cancers and Brain Tumors Using Knowledge-Based Planning Models

**DOI:** 10.3390/cancers18142216

**Published:** 2026-07-09

**Authors:** Marzena Janiszewska, Tomasz Siudziński, Krzysztof Składowski, Adam J. Maciejczyk

**Affiliations:** 1 Lower Silesian Oncology, Pulmonology and Hematology Center, 53-413 Wroclaw, Poland; 2 1st Radiation and Clinical Oncology Department, Maria Sklodowska-Curie National Research Institute of Oncology, 44-102 Gliwice, Poland; 3 Department of Radiotherapy, Lower Silesian Oncology, Pulmonology and Hematology Center, 53-413 Wroclaw, Poland; 4 Department of Oncology, Wroclaw Medical University, 53-413 Wroclaw, Poland

**Keywords:** knowledge-based planning, RapidPlan, VMAT, head and neck cancers, brain tumors, radiotherapy QA, outliers, monitor units, Kolmogorov–Smirnov test

## Abstract

**Objectives:** Our objective was to develop and evaluate a locally trained knowledge-based planning (KBP) model for head and neck (H&N), brain, and central nervous system malignancies using RapidPlan, and to determine whether standard statistical metrics such as the coefficient of determination (R^2^) and outlier frequency are definitive predictors of clinical utility. **Methods:** An institutional dataset of 594 plans was retrospectively curated into a 497-plan training set. Performance was evaluated in 370 paired plan comparisons generated with identical beam geometry. Training–validation overlap was explicitly quantified at both plan and patient levels, and a plan-level held-out sensitivity analysis was performed. Additional analyses included monitor units (MUs), subgroup assessment, clinically relevant OAR threshold achievement, and 95% confidence intervals for paired differences. **Results:** The validation set included 370 plans from 289 patients. At the plan level, 303 validation plans overlapped with the training model, and 67 were held-out cases; at the patient level, no fully patient-independent validation cohort was available. RapidPlan maintained target coverage while reducing OAR doses, including oral cavity Dmean (−7.62%Rx; 95% CI: −8.91 to −6.32; *p* < 0.001) and larynx Dmean (−7.57%Rx; 95% CI: −9.14 to −6.00; *p* < 0.001). The same direction of benefit was observed in the plan-level held-out subset. MU did not increase with RapidPlan and decreased from 764.2 ± 275.5 to 695.8 ± 210.3 MU (Delta = −68.4 MU; 95% CI: −89.4 to −47.4; *p* < 0.001). **Conclusions:** A high R^2^ was not required for clinically useful optimization objectives in this heterogeneous cohort. However, the retrospective design and patient-level overlap limit claims of full generalizability. The model should therefore be interpreted as a clinically useful standardization and decision-support tool requiring expert review rather than as a replacement for the judgment of physicists and radiation oncologists.

## Simple Summary

Developing automated radiation treatment plans for complex head and neck cancers and brain tumors remains a major challenge due to anatomical variability and differences in clinical planning practice. This study evaluates a single institutional knowledge-based planning model designed to standardize and streamline treatment planning across connected radiotherapy sites. The findings show that conventional model-quality statistics do not always predict clinical usefulness. With careful curation of the training data, the automated system maintained target coverage while reducing doses to several organs at risk, including the oral cavity and larynx. The model should be interpreted as a planning standardization and decision-support tool that reduces manual variability, while final plan approval remains the responsibility of experienced physicists and radiation oncologists.

## 1. Introduction

Planning Intensity-Modulated Radiation Therapy (IMRT) and Volumetric Modulated Arc Therapy (VMAT) are multidimensional processes in which substantial variability in dose distribution to organs at risk (OARs) can persist even when identical clinical criteria are applied [1]. This inconsistency often stems from a combination of the planner’s experience, institutional “unwritten rules” regarding optimizer use, and interobserver differences in contouring and target volume definition [2,3]. To address these challenges, there is an increasing shift toward the automation and standardization of treatment planning through knowledge-based planning (KBP), which leverages libraries of previously approved plans to predict achievable dose constraints and generate patient-specific optimization objectives [4,5,6,7,8].

As a proven tool for radiotherapy automation, the RapidPlan commercial KBP system (Varian Medical Systems) utilizes data-driven workflows to facilitate rapid automated treatment planning. Rather than employing deep neural networks, this regression-based architecture applies principal component analysis and mathematical modeling to map the direct spatial relationships between patient geometry and dose–volume histogram (DVH) parameters [9]. The algorithm stratifies OARs into regions based on their spatial relationship to the planning target volume (PTV) and beam geometry, specifically identifying overlap, in-field, and out-of-field regions, to distinguish areas that can be actively controlled during optimization from those dominated by scatter and transmission [10,11,12].

The clinical necessity for such precision is paramount in head and neck (H&N) cancers, a region characterized by the most complex anatomy in the human body. The dense concentration of critical structures within small volumes, often in proximity to target volumes, demands a high degree of accuracy in assessing disease topography and microscopic spread. Standard treatment protocols, whether definitive or adjuvant, require delivering high therapeutic doses (typically 60–70 Gy) to the gross tumor volume (GTV) while strictly limiting exposure to OARs, such as the salivary glands and oral cavity, to preserve patient quality of life [13,14]. Achieving this balance represents the core challenge for the physicist, traditionally relying on practical experience but increasingly supported by KBP strategies.

In practice, the quality of a RapidPlan model is frequently evaluated through geometric dose-fit measures, such as the coefficient of determination (R^2^). However, predictive performance and generalizability are also influenced by data consistency and outlier management [15,16,17,18]. In the H&N context, population heterogeneity and variability in clinical presentation can significantly impact model behavior [15,16,18,19]. Our institution implemented RapidPlan in 2016 during a period of rapid VMAT expansion. Early development revealed that model statistics could highlight sources of variation often obscured in routine practice, including inconsistent optimization strategies and variability in radiation oncologists’ contouring practices.

This clinical environment is characterized by a highly integrated infrastructure comprising ten Varian linear accelerators deployed across three connected locations: the main facility, which operates six accelerators, and two satellite radiation therapy centers situated approximately 100 km away, each equipped with two accelerators. This entire network operates seamlessly on a shared Eclipse treatment planning system and a unified Aria oncology information system. Managing an interconnected configuration of ten accelerators across multiple geographic locations under a single planning framework represents a unique operational paradigm. By utilizing a single computational model across all three facilities, the institution ensures absolute clinical consistency and significantly minimizes the likelihood of dosimetric adverse events. Furthermore, building a unified RapidPlan model for a broad, comprehensive cohort of diverse H&N clinical cases completely mitigates the latent risk of selecting or applying an inappropriate model to a specific patient scenario.

Given the organizational structure of our institution, which includes three satellite sites and multiple clinical teams, immediate standardization of historical data was not feasible, and population heterogeneity was considered unavoidable. In many published RapidPlan implementations, model development is intentionally restricted to narrower, site-specific indications in order to reduce anatomical and dosimetric variability; for example, Fogliata et al. developed a model focused on nasopharyngeal, oropharyngeal, and hypopharyngeal cancers [20]. Such stratification is methodologically justified and may improve geometric homogeneity. However, in a multi-site clinical network, maintaining multiple narrowly defined models also introduces practical challenges, including the need for consistent model selection across planners, sites, and complex cases with overlapping anatomical regions. Therefore, our approach was deliberately pragmatic: rather than attempting to prove the dosimetric superiority of a unified model over several site-specific models, we aimed to evaluate whether a single broad anatomical model could provide clinically acceptable and reproducible planning support across the full spectrum of institutional H&N, brain, and CNS cases.

To address this gap, we adopted a pragmatic approach: rather than fragmenting our database, we developed a single, broad anatomical model that extends the traditional H&N region to incorporate brain cancers within a unified training repository using standardized OAR nomenclature. While geometric outliers were accepted as reflecting natural anatomical diversity, dosimetric outliers were actively controlled to reduce prediction variance. This study describes the development and evaluation of a locally trained RapidPlan model designed to ensure consistency and reproducibility across multiple sites. We specifically investigate whether a high R^2^ value is a strictly necessary condition for stable DVH predictions and how a high proportion of outliers should be interpreted when substantial improvements in dosimetric metrics are still achieved. This shifts the focus from simply proving the superiority of KBP over manual planning to identifying which quality metrics most accurately reflect clinical utility in a heterogeneous environment.

## 2. Materials and Methods

**Figure 1:**
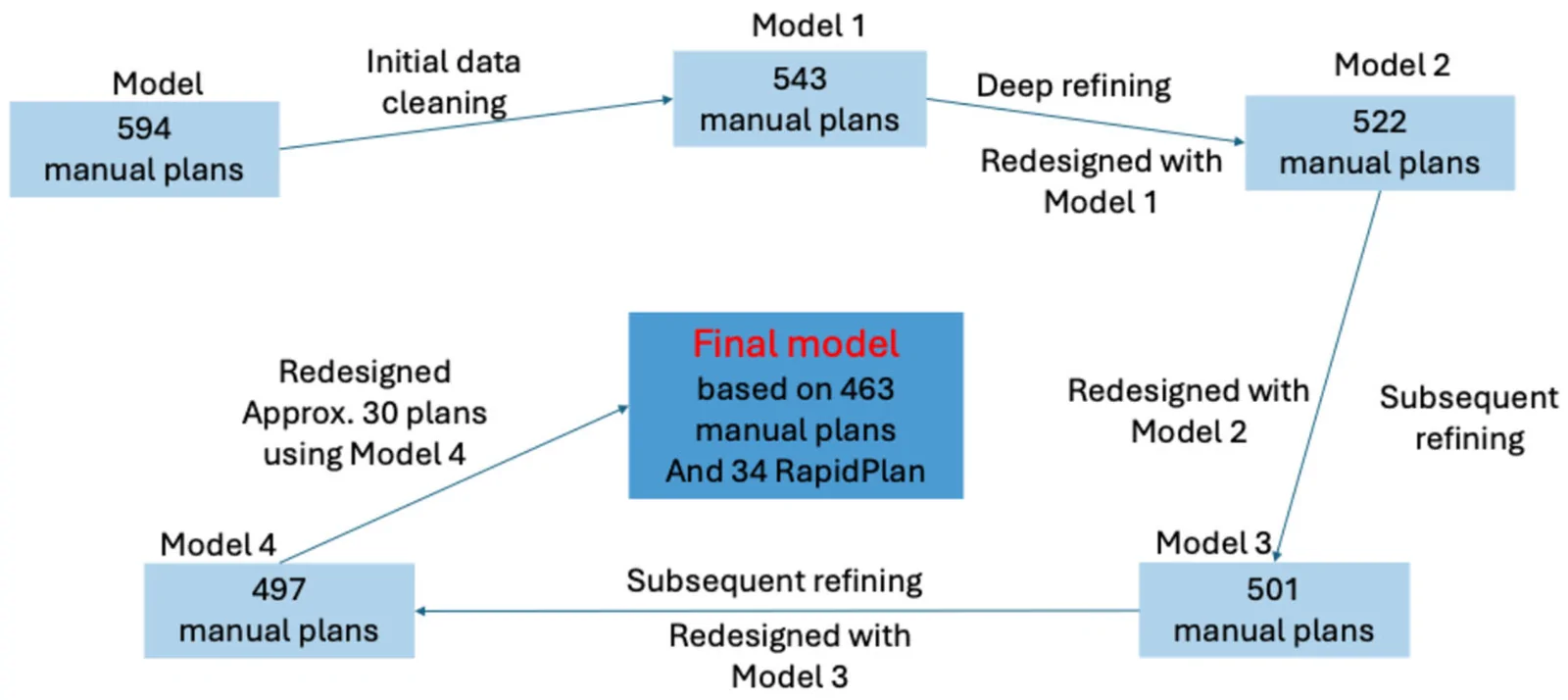
Flowcharts of the KBP (Knowledge-Based Planning) model training workflow for H&N and brain cancers based on an iterative data selection approach. A multi-step data curation approach was applied to the cases to optimize the training database while preserving anatomical variability. Manual plans are treatment plans manually generated by medical physicists. The stages represent the transition from the initial dataset to the final model through successive phases of data refining and outlier removal.

This retrospective study focused on the systematic construction and validation of a KBP model for H&N, brain, and CNS tumors. Database curation was performed in a staged manner, starting from an initial pool of 594 plans and progressing through multiple iterations of data cleaning and refinement (Figure 1). The final model was trained on 497 plans, comprising 463 original clinical plans and 34 refined plans re-optimized using earlier iterations of the model. Model performance was evaluated across 370 plan pairs by comparing original clinical plans against retrospectively generated KBP plans created with identical beam geometry. Because this was a retrospective model-development study, the overlap between training and validation data was explicitly quantified at both plan and patient levels rather than assumed to be absent.

All plans were calculated within the Eclipse treatment planning system (Varian Medical Systems, Palo Alto, CA, USA) using the Acuros XB algorithm, version 15.6. To ensure a direct comparison of optimization quality, each RapidPlan was generated using the same beam geometry and arc arrangement as its corresponding clinical counterpart. This methodology ensured that any observed dosimetric differences resulted strictly from the optimization objectives and constraints rather than variations in beam delivery.

The model utilized a unified database approach, incorporating a diverse range of diagnoses, multiple planning target volumes (PTV1–PTV4), and various fractionation schemes. This institutional strategy favors a comprehensive dataset over fragmented models to better capture complex anatomical and dosimetric correlations. The cohort included a broad spectrum of clinical sites categorized by ICD-10 classifications, including: Malignancies of the lip (C00), base of tongue (C01), dorsal surface of tongue (C02), gingiva (C03), floor of mouth (C04), palate (C05), other and unspecified parts of the oral cavity (C06), parotid gland (C07), other and unspecified major salivary glands (C08), tonsil (C09), oropharynx (C10), nasopharynx (C11), pyriform sinus (C12), hypopharynx (C13), other and ill-defined sites of the lip, oral cavity, and pharynx (C14), nasal cavity and middle ear (C30), accessory sinuses (C31), larynx (C32), thymus (C37), and eye and adnexa (C69). To further expand the model’s scope, we extended our study to include traditional head and neck malignancies, as well as primary central nervous system tumors of the meninges (C70) and brain (C71). Including this diverse array of clinical diagnoses is grounded in the underlying mechanics of mathematical planning algorithms. For machine learning systems operating in radiation therapy, the primary driver of predictive accuracy is not the oncology classification itself, but the direct spatial correlation and geometric relationship between unambiguously named critical structures and target volumes within the active beam path. Because the RapidPlan system characterizes these structural relationships using principal component analysis and regression techniques to estimate dose–volume histograms, combining overlapping anatomical zones, such as brain and central nervous system tumors alongside traditional head and neck sites, allows the model to leverage a much larger pool of shared spatial features. This broad inclusion directly increases the dataset’s statistical power, enabling the generation of robust, data-driven optimization objectives within a single, unified framework.

The model was developed using a wide spectrum of radiation dose regimens, including simultaneous integrated boost (SIB) and conventional fractionation approaches across radical and palliative settings. The following dose-fractionation schedules were utilized for the four PTV levels:

PTV1: 20 Gy in 5 fractions; 30 Gy in 10 fractions; 40.5 Gy in 15 fractions; 62.5 Gy in 25 fractions; 45 Gy in 18 fractions; 65.25 Gy in 29 fractions or 63 Gy in 28 fractions; 69.96 Gy in 33 fractions; 70 Gy in 35 fractions; 60 Gy in 30 fractions; 54 Gy in 27 fractions; 50 Gy in 25 fractions; 10 Gy in 5 fractions; 6 Gy in 3 fractions; and 36 Gy in 20 fractions.

PTV2: 66 Gy in 33 fractions; 61.05 Gy in 33 fractions; 59.4 Gy in 33 fractions or 54 Gy in 30 fractions; 62.9 Gy in 37 fractions or 56.1 Gy in 33 fractions; and 54 Gy in 33 fractions.

PTV3: 61.05 Gy in 33 fractions (1.85 Gy/fraction); 59.4 Gy in 33 fractions or 54 Gy in 30 fractions; 62.9 Gy in 37 fractions or 56.1 Gy in 33 fractions; and 54 Gy in 33 fractions.

PTV4: 56.1 Gy in 33 fractions; and 54.1 Gy in 33 fractions.

A total of 24 organ-at-risk (OAR) types were included; bilateral structures (e.g., parotids) were mapped to single model entries to maintain consistency.

Built-in RapidPlan qualitative statistical tools guided model training. The coefficient of determination (R^2^) was employed to assess regression accuracy in the in-field regions. Outlier detection was performed using a multi-parametric approach: geometric outliers were identified via modified Z-scores (mZ); dosimetric outliers were flagged using studentized residuals (SR) and areal differences (dA); and influential points were identified using Cook’s distance (CD).

Dosimetric metrics were normalized as a percentage of the prescribed dose (%Rx). For every patient, the difference was calculated as Delta (Δ) = (RapidPlan − Clinical Plan). For OARs, negative delta values indicated superior sparing by the KBP model. Statistical significance was determined using a paired Student’s *t*-test, while the Kolmogorov–Smirnov test evaluated the agreement of distributions.

A binary “better plan” analysis determined the frequency with which the KBP model outperformed clinical plans. For OARs, lower Dmean or Dmax values were considered favorable; for PTVs, higher D98% and lower D2% were considered favorable, and lower standard deviation (SD%) was considered favorable. Composite patient-level indices were calculated only from structures present in each patient and paired between the clinical and RapidPlan plans. Each available structure/metric contributed with equal weight, and missing structures were not imputed. To keep interpretation consistent, signs were oriented so that positive OAR composite values indicated a RapidPlan advantage, whereas for PTVs, the direction of improvement was defined separately for coverage, hotspot reduction, and dose homogeneity.

### 2.1. Training–Validation Overlap, Cohort Characterization, and Sensitivity Analyses

The final training set contained 497 plans from 401 unique patients, while the validation cohort contained 370 plan pairs from 289 unique patients. At the plan level, 303 validation plans (81.9%) were included in the final training model, whereas 67 (18.1%) were held out. Assessment of the training cohort revealed that 194 plans were utilized exclusively for model training. At the patient level, all 289 validation patients had at least one plan represented in the training model; consequently, a fully patient-independent validation cohort could not be established. This critical distinction between plan-level and patient-level independence was strictly maintained during the interpretation of all subsequent results.

The validation plans originated from three institutional sites: Wroclaw (*n* = 295), Jelenia Gora (*n* = 43), and Legnica (*n* = 32). Eight medical planners contributed to the original clinical plans: Planner A (*n* = 73), Planner B (*n* = 72), Planner C (*n* = 69), Planner D (*n* = 58), Planner E (*n* = 52), Planner F (*n* = 36), Planner G (*n* = 8), and Planner H (*n* = 2). The original clinical plans were not categorically reclassified as clinically unacceptable. Instead, potentially suboptimal entries for model training were operationally identified as cases requiring exclusion or outlier management during the model curation phase, resulting in the exclusion of 97 of the initial 594 candidate plans.

Among the 34 refined plans included in the final training library, 18 were present within the validation cohort and belonged to the plan-level overlap subset. These cases were explicitly accounted for within the cohort description, whereas the plan-level held-out subset excluded them by definition. Sensitivity analyses were performed within the plan-level held-out subset using the same paired statistical approach applied to the full validation cohort. This subset strictly excluded all plans present in the final training model.

### 2.2. Plan Complexity and Deliverability

To evaluate whether dosimetric gains occurred at the expense of increased treatment complexity, total monitor units (MUs) were extracted for each clinical and RapidPlan plan. The total MUs were calculated as the sum of meterset values for therapeutic beams only, excluding setup and imaging fields. Because the RapidPlan plans were generated retrospectively and not delivered clinically, paired patient-specific Portal Dosimetry gamma measurements were unavailable for the RapidPlan cohort. Portal Dosimetry results were exclusively available for the clinically delivered manual plans; consequently, unpaired gamma reporting was deemed an invalid basis for comparison. Monitor units were therefore adopted as the primary paired metric for complexity analysis. Formal modulation complexity metrics, such as MCS or MCSv, were not available in the retrospective dataset; therefore, total MUs were used as an available paired surrogate of plan complexity rather than as a direct replacement for MCS. The absence of paired gamma verification and formal modulation complexity analysis is acknowledged as a study limitation.

### 2.3. Subgroup and Clinically Relevant Threshold Analyses

Subgroup analyses were performed for H&N versus brain/CNS cases and for the three institutional sites. In addition to mean dose differences, clinically interpretable threshold analyses were added for selected OARs. Oral cavity Dmean < 40 Gy and larynx Dmean < 45 Gy were evaluated at the plan level after conversion from %Rx to Gy. Additional structure-specific thresholds were evaluated for non-parotid salivary glands (Dmean < 26 Gy), lenses (Dmax < 10 Gy), eyes (Dmean < 38 Gy), and eyes (Dmax < 45 Gy). Bilateral structures were analyzed per structure instance when both manual and RapidPlan values were available.

## 3. Results

A comprehensive summary of the model’s qualitative statistics is presented in Table 1. A high percentage of cases were flagged as outliers; however, these predominantly comprised geometric outliers. Dosimetric deviations were largely mitigated during the iterative training process. Consequently, the observed outlier frequency primarily reflects the inherent variability in the geometry–beam–target relationship and the diversity of institutional contouring rather than a high prevalence of suboptimal dosimetry within the training set. R^2^ for OARs exhibited significant variability. While some structures achieved high fits (e.g., the brain), others demonstrated moderate R^2^ values, such as the oral cavity (R^2^ = 0.54; 30% outliers). Our experience suggests that the outlier percentage alone is an insufficient predictor of clinical utility. Instead, the clinical efficacy of a model must be determined by juxtaposing these statistical indicators with actual dosimetric performance in real-world validation cases.

Target coverage parameters were generally comparable between RapidPlan and original clinical plans (Table 2). Differences in D98% were not statistically significant for PTV1 (*p* = 0.423) and borderline for PTV2 (*p* = 0.055), whereas PTV3 and PTV4 showed small, statistically significant decreases in %Rx (*p* = 0.031 and *p* = 0.019, respectively). In absolute-dose terms, the mean differences were −0.20 Gy for PTV3 D98% and −0.29 Gy for PTV4 D98%, indicating limited clinical magnitude. In the plan-level held-out subset, the absolute D98 differences for PTV3 and PTV4 were not statistically significant: PTV3 D98 decreased by −0.28 Gy (95% CI: −0.64 to 0.07; *p* = 0.114), and PTV4 D98 decreased by −0.53 Gy (95% CI: −1.30 to 0.24; *p* = 0.157).

The model demonstrates that moderate R^2^ values do not preclude clinical utility. The oral cavity provides the most prominent evidence: despite an R^2^ of 0.54, RapidPlan achieved a substantially lower mean dose than clinical plans (Delta = −7.62%Rx; 95% CI: −8.91 to −6.32; *p* < 0.001). A similar trend was observed for the larynx (Delta = −7.57%Rx; 95% CI: −9.14 to −6.00; *p* < 0.001), salivary glands (Delta = −4.36%Rx; *p* < 0.001), thyroid (Delta = −5.29%Rx; *p* < 0.001), and lips (Delta = −6.98%Rx; *p* < 0.001). For serial organs, interpretation was focused primarily on Dmax rather than Dmean. RapidPlan showed a significant reduction in brainstem Dmax (Delta = −2.14%Rx; *p* < 0.001) and brain Dmax (Delta = −5.80%Rx; *p* < 0.001), while spinal cord Dmax remained statistically neutral (Delta = −0.10%Rx; 95% CI: −1.03 to 0.83; *p* = 0.833).

Cohort composition and training–validation overlap are summarized in Table 3. Plan-complexity results are summarized in Table 4. Clinically relevant threshold-achievement results are summarized in Table 5.

Within the plan-level held-out subset (n = 67), the principal OAR endpoints demonstrated a consistent directional benefit. Oral cavity Dmean changed by Delta = −8.84%Rx (95% CI: −12.74 to −4.94; *p* < 0.001), larynx Dmean by Delta = −7.24%Rx (95% CI: −11.86 to −2.62; *p* = 0.003), brain Dmax by Delta = −4.43%Rx (*p* = 0.011), thyroid Dmean by Delta = −5.60%Rx (*p* < 0.001), and lips Dmean by Delta = −5.61%Rx (*p* = 0.006). Treatment complexity also significantly decreased (Delta = −72.2 MU; 95% CI: −121.1 to −23.3; *p* = 0.004). Similarly, threshold analysis showed higher compliance rates for oral cavity Dmean < 40 Gy (92.9% to 100.0%), larynx Dmean < 45 Gy (77.8% to 85.2%), and non-parotid salivary gland Dmean < 26 Gy (75.9% to 84.3%).

Because OAR availability differed substantially between H&N and brain/CNS cases, subgroup OAR analysis was interpreted only for structures present and paired within each subgroup, rather than as an identical structure-by-structure comparison. In the H&N subgroup, RapidPlan reduced oral cavity Dmean by −7.64%Rx, larynx Dmean by −7.77%Rx, salivary gland Dmean by −4.25%Rx, thyroid Dmean by −4.96%Rx, mandible Dmax by −1.53%Rx, brainstem Dmax by −2.91%Rx, brain Dmax by −8.64%Rx, and ear Dmax by −4.25%Rx. Spinal cord Dmax, lens Dmax, and eye Dmax remained statistically neutral. In the brain/CNS subgroup, RapidPlan reduced brain Dmax by −1.63%Rx, optic nerve Dmax by −2.76%Rx, eye Dmax by −3.10%Rx, ear Dmax by −8.45%Rx, and pituitary Dmax by −8.64%Rx. Brainstem Dmax, optic chiasm Dmax, and spinal cord Dmax were statistically neutral, while lens Dmax showed a small increase of +0.45%Rx. Overall, the subgroup analysis supported a generally favorable direction of OAR-dose changes among available structures, but not uniform improvement for every OAR in both anatomical subgroups.

**Figure 2:**
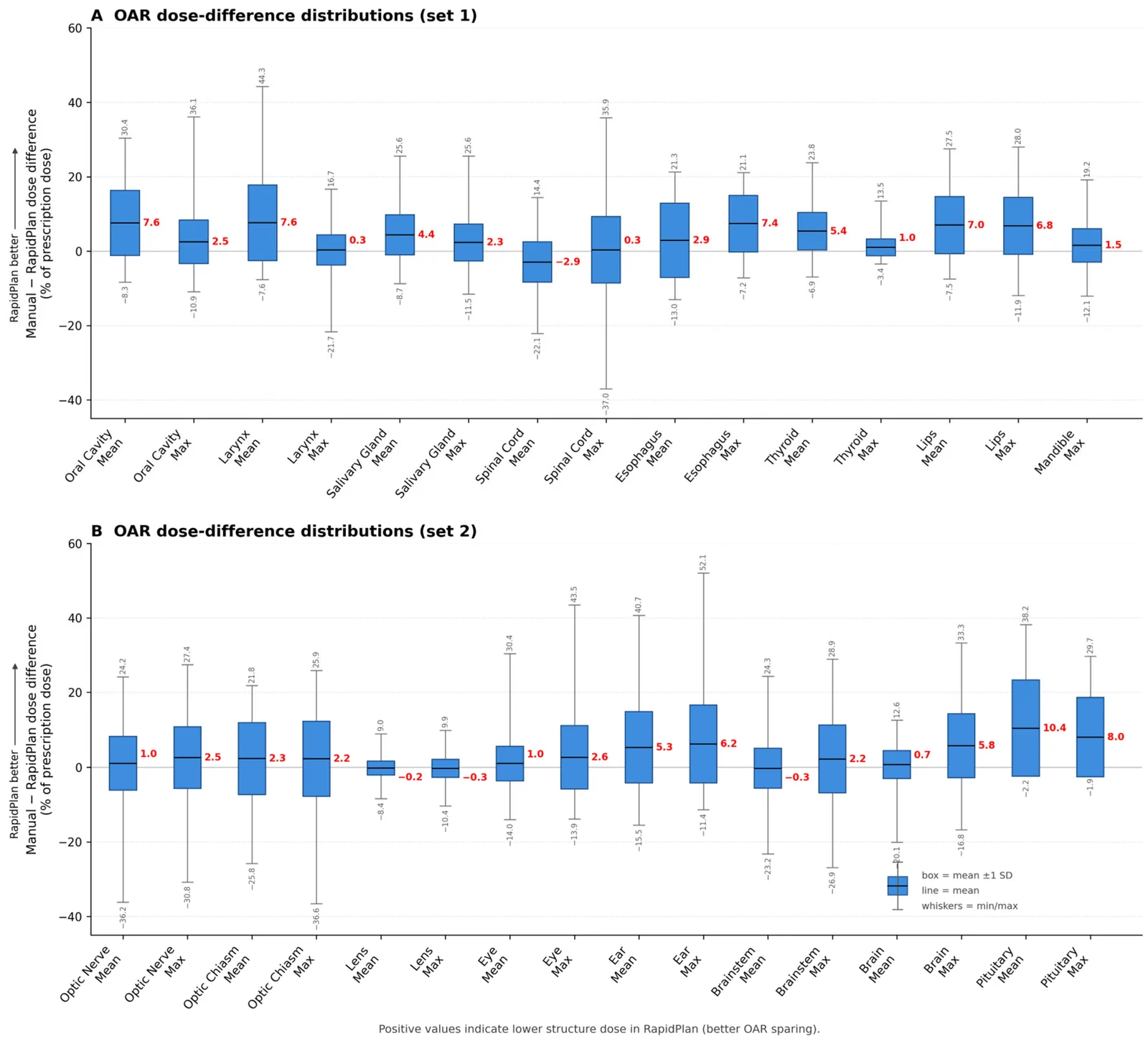
OAR dose-difference distributions. Values are Manual − RapidPlan (%Rx); positive values indicate lower OAR dose with RapidPlan. Boxes show mean ± 1 SD, central lines the mean, whiskers the min–max range, and red labels the mean.

**Figure 3:**
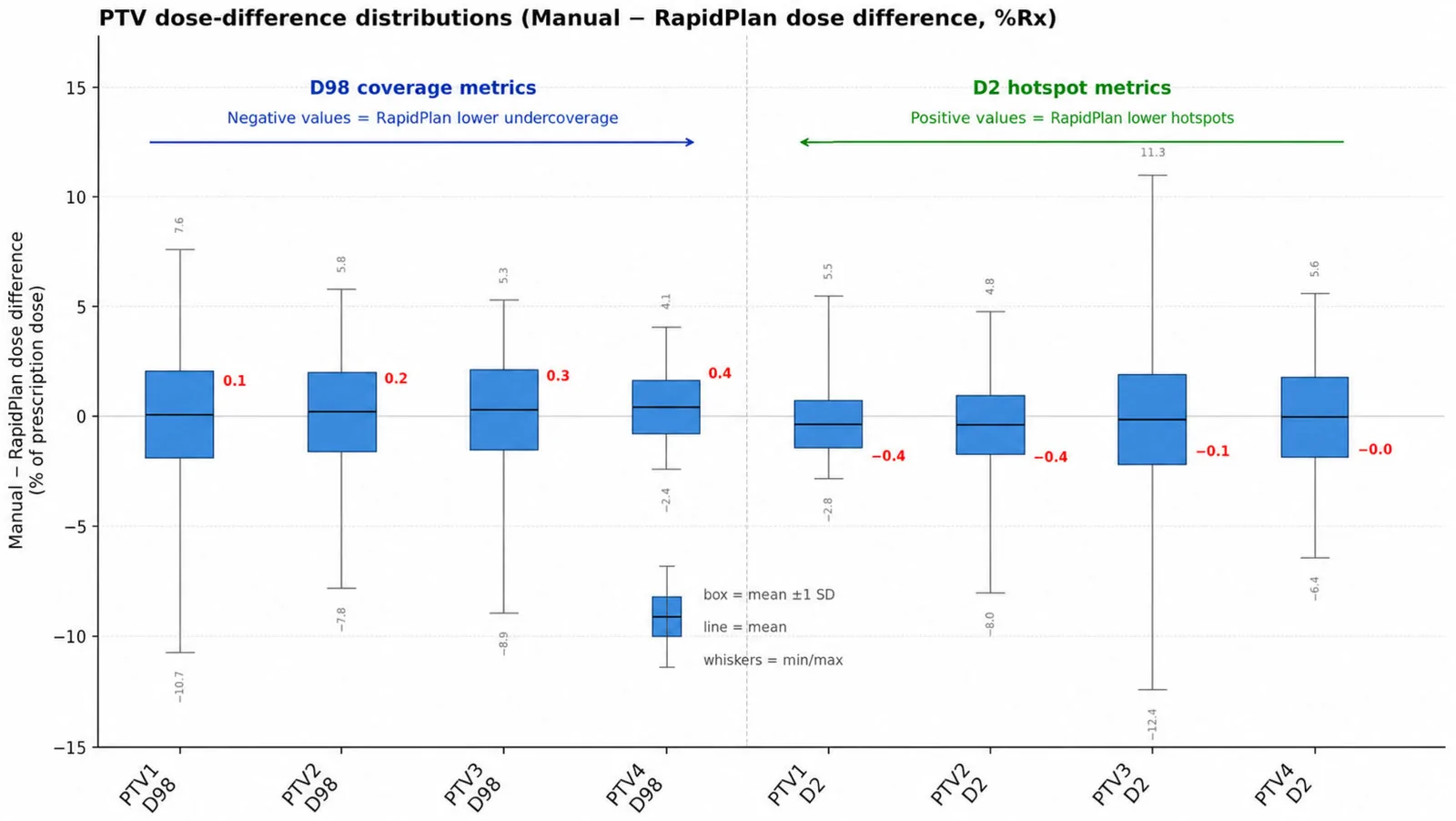
PTV dose-difference distributions. Values are Manual − RapidPlan (%Rx). For D98%, positive values indicate lower undercoverage with RapidPlan; for D2%, negative values indicate lower hotspots with RapidPlan. Boxes show mean ± 1 SD, central lines the mean, whiskers the min–max range, and red labels the mean.

**Figure 4:**
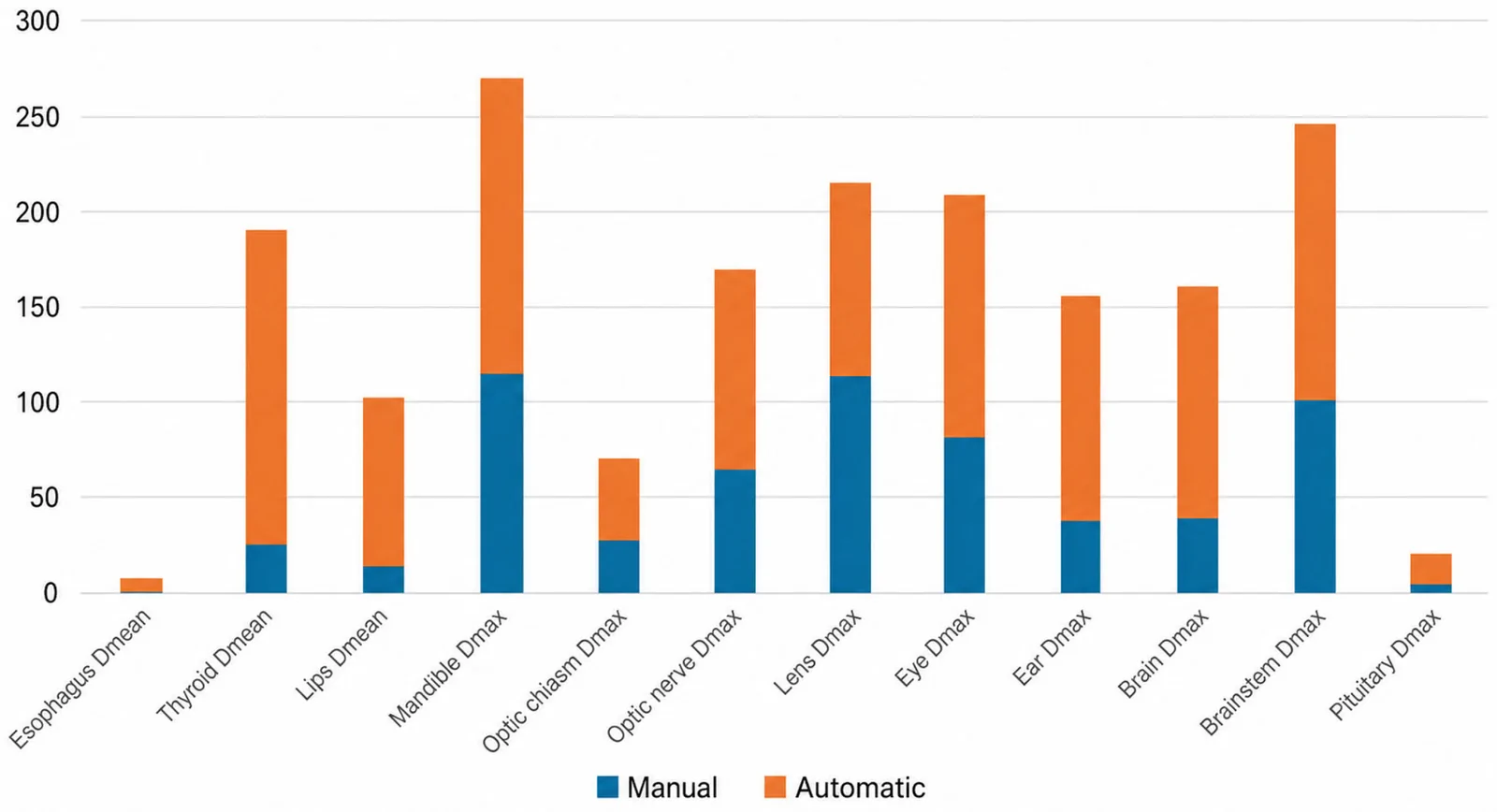
Binary comparison of dosimetric performance in radiotherapy planning. Grouped bars show the number of paired observations in which manual planning or RapidPlan achieved the superior metric value. Bilateral OARs are combined as left and right paired observations.

**Figure 5:**
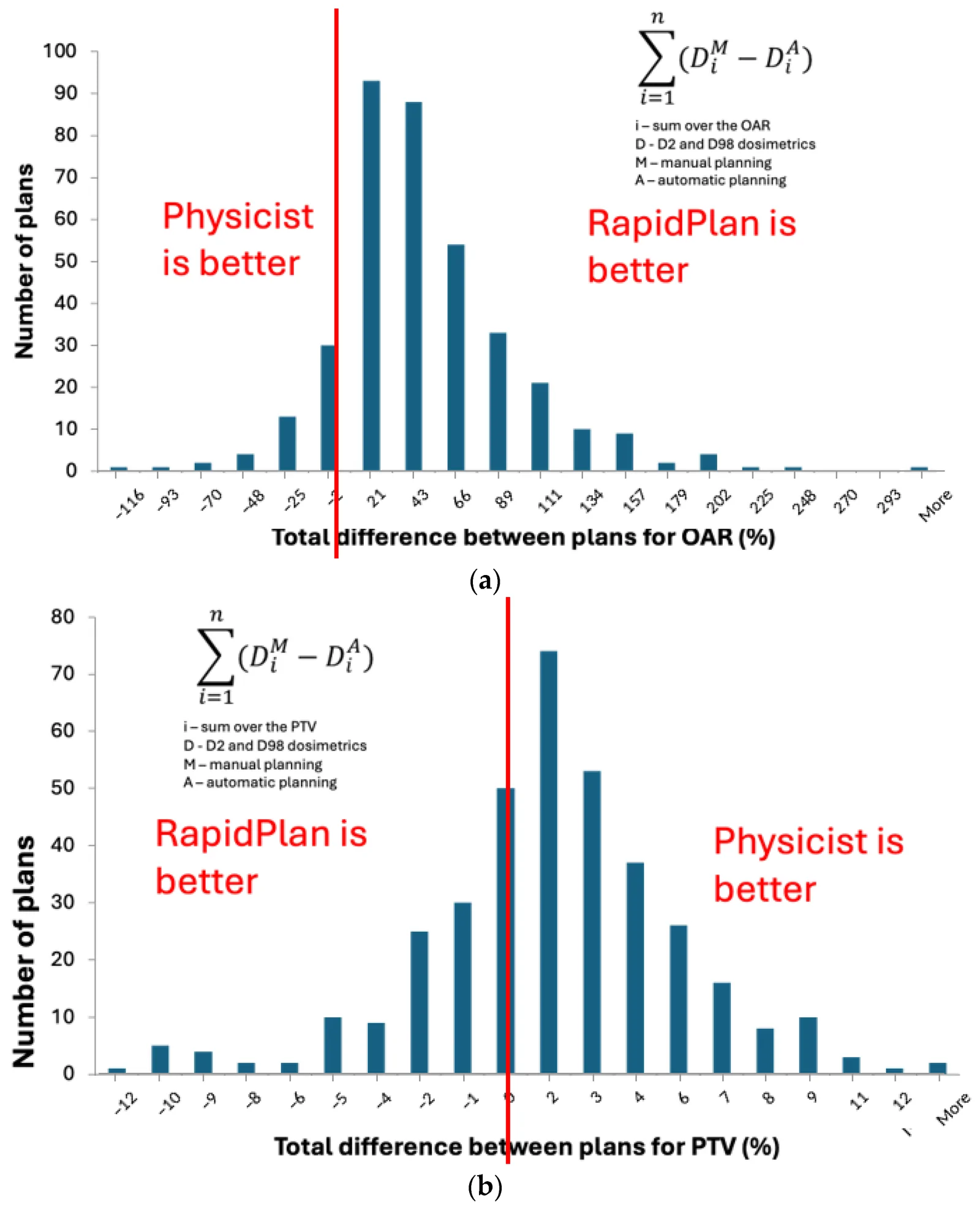
Distribution of the composite OAR (**a**) and PTV (**b**) indexes at the individual patient level. The index represents the sum of differences in D2 and D98 metrics between manual and automated plans. a. For OAR, values > 0 indicate a dosimetric advantage for RapidPlan, while values < 0 indicate an advantage manual planning by a physicist. b. For PTV, values > 0 indicate a dosimetric advantage for manual planning by a physicist, while values < 0 indicate an advantage for the automated RapidPlan.

The Dmean (%Rx) distributions for OARs (Figure 2, Figure 3, Figure 4 and Figure 5) revealed a systematic shift toward lower values in the RapidPlan cohort, suggesting a population-wide improvement in sparing rather than isolated successes. Additionally, several structures showed reduced dose spread, indicating enhanced reproducibility.

Figure 2 and Figure 3 illustrate the distribution of paired dose differences, including the mean, ±1 SD range, and the observed minimum and maximum values. Evidence of planning standardization is primarily found in the direct comparison of standard deviations (SD) between the two groups. As shown in Table 2, for many Dmean-based metrics, the SD was lower in RapidPlan than in clinical plans (e.g., oral cavity: 13.5 vs. 17.4; lips: 17.5 vs. 21.6), indicating superior consistency across the patient population. This pattern was less pronounced for Dmax metrics, likely due to the higher sensitivity of maximum doses to specific anatomical variations.

The Kolmogorov–Smirnov test confirmed that, for several structures, differences existed not only in mean values but in the overall shape of the dose distributions. This suggests that KBP influences the entire optimization trajectory rather than just isolated DVH points. Binary analysis shown in Figure 4 and composite indices shown in Figure 5a,b further confirms the model’s consistent advantage across multiple OARs. At the individual patient level, the summed difference for OARs was predominantly positive (Figure 5a), indicating that RapidPlan more frequently achieves a superior global dose distribution. Conversely, the composite index for PTVs remained centered near zero (Figure 5b), reinforcing the observation that the model maintains target coverage while primarily enhancing the predictability and efficiency of OAR sparing.

## 4. Discussion

The integration of RapidPlan models into H&N, brain, and central nervous system radiotherapy supports the transition from exclusively manual optimization toward a more standardized and reproducible planning workflow. In this study, KBP-generated plans maintained clinically comparable target coverage under identical arc geometries while reducing dose to multiple OARs. These findings support the use of KBP as a planning standardization and decision-support tool; however, final plan acceptance remains dependent on expert review by medical physicists and radiation oncologists.

A noteworthy aspect of this study is the deliberate inclusion of a highly heterogeneous patient cohort. While the existing literature limits KBP model construction to narrow clinical indications, such as creating separate models specifically for the larynx, tonsils, or salivary glands, our approach favored a comprehensive, broad anatomical model [9,12,20]. This heterogeneity extended even to the inclusion of brain cancer cases, which, although strictly not H&N tumors, share overlapping anatomical regions and critical structures. By incorporating diverse anatomical sites, varied target volumes, and different dose regimens into a single framework, we have developed a more robust and user-friendly tool. This emphasizes that a well-curated KBP model can maintain high performance across a broad clinical spectrum, rather than being restricted to a specific sub-site.

Beyond the documented capacity of RapidPlan to reduce OAR doses [21,22,23,24], our findings highlight a more nuanced interpretation of model statistics. We specifically evaluated the acceptable threshold for outliers, the impact of database quality assurance on prediction stability, and whether high R^2^ values are truly indicative of reliable, clinically valid optimization objectives. Our results indicate that a high R^2^ value is not a prerequisite for clinical utility. This aligns with recent findings suggesting that R^2^ primarily reflects the geometric-to-DVH parameterization fit rather than the model’s ability to drive the optimizer toward an ideal target–OAR trade-off [25]. For instance, the oral cavity achieved statistically significant reductions in mean dose despite moderate regression fits and a high frequency of outliers. Conversely, our internal prostate model yielded high R^2^ values despite similar outlier rates. Therefore, R^2^ should be viewed as a supportive metric rather than a definitive benchmark of model quality.

The implementation of this model significantly enhances dosimetric consistency. Lower standard deviations in mean dose metrics across the RapidPlan cohort suggest that the model effectively levels inter-patient variability by neutralizing the impact of individual planning habits and random errors, supporting existing research [4,26,27]. While this consistency was less pronounced for maximum dose metrics, which remain highly sensitive to patient-specific anatomy, the model successfully streamlines planning by automating constraint entry. Although PTV1 and PTV2 coverage was maintained, the slight decrease in D98% for PTV3 and PTV4 highlights the necessary trade-off between aggressive OAR sparing and secondary target coverage.

Our analysis further demonstrates that a high frequency of outliers, particularly those driven by geometric variability, does not inherently compromise model utility. However, clinical validity remains contingent upon the active management of the training library. This reinforces the “data integrity” principle emphasized by Kaderka et al., noting that predictive accuracy reflects the dosimetric excellence of training cases rather than mere database volume [28]. In this framework, it is vital to distinguish between geometric outliers, which reflect natural anatomical diversity, and dosimetric outliers, which signify inconsistent priorities or lower-quality legacy plans [17]. Following our curation protocols, geometric outliers predominated while dosimetric outliers were significantly reduced, ensuring that the model reflects the highest achievable clinical standards rather than a mere average of past performance.

The success of the H&N model is rooted in a rigorous two-pronged data refinement strategy that integrates staged database cleaning with selective replanning. This optimization prioritizes high-quality dosimetry over historical outliers, reinforcing the principle that KBP is a dynamic tool requiring active quality control. For centers implementing KBP, it is essential to distinguish between geometric outliers and dosimetric outliers. This emphasis on refinement aligns with the recent literature suggesting that traditional performance metrics should not serve as the sole indicators of model quality without rigorous outlier management [17]. Ultimately, whether a facility opts for selective replanning or a comprehensive database overhaul, the goal remains ensuring that the model reflects superior clinical standards.

Relative to the existing literature, our findings illustrate how a KBP model functions not only as an automated tool but also as a mechanism for quality assurance [29]. A robust evaluation of a RapidPlan model should incorporate three distinct primary elements. First, one must consider the statistical context of outliers, where the nature of geometric or dosimetric deviations is interpreted relative to the training dataset volume. Second, the focus shifts to clinical relevance, requiring that model performance be assessed in structures with moderate R^2^ values. These often represent critical quality-of-life organs which demand high precision despite statistical variability. Third, the model addresses practical execution through the precise configuration of optimization objectives, emphasizing how the distinction between Dmean and Dmax in serial organs dictates which clinical metrics can realistically be improved [30].

Despite these advancements, this study has several limitations. First, its retrospective nature and use of identical arc geometry across both cohorts isolated the impact of optimization objectives but precluded the assessment of potential gains from geometry modification. Second, although 67 validation plans were held out at the plan level, all validation patients were represented in the training population; thus, the study lacks a fully patient-independent external validation cohort. Third, because the retrospectively generated RapidPlan plans were not clinically delivered, paired Portal Dosimetry gamma measurements were unavailable. Consequently, the plan complexity assessment was limited to MU, whereas prospective implementation should incorporate paired patient-specific QA, gamma analysis, and ideally formal complexity metrics such as MCS.

Another limitation is that the originally delivered clinical plans were not retrospectively reclassified as clinically acceptable or unacceptable using a formal institutional scoring system. Therefore, suboptimal training entries were defined operationally as cases requiring exclusion, outlier management, or selective replanning during model curation, rather than as clinically unacceptable plans.

The present analysis was restricted to photon EBRT planning utilizing VMAT/IMRT within Eclipse/RapidPlan and was not designed to compare photon EBRT with proton therapy or brachytherapy/interventional radiotherapy. These modalities differ substantially in planning technique, dose calculation, delivery, availability, and clinical indications; therefore, the results of this RapidPlan model should not be extrapolated to them. Comparative evaluations between EBRT, proton therapy, and brachytherapy should be addressed in separate, modality-specific studies.

## 5. Conclusions

The RapidPlan H&N/brain model serves as a consistent generator of high-quality optimization objectives, reducing manual variability while preserving the need for expert oversight. Meaningful OAR sparing was achieved despite high geometric variability and moderate R^2^ values, provided that the training library was maintained with strict dosimetric integrity. The unified model reduced the operational risk of selecting an inappropriate site-specific model and showed consistent MU-based complexity trends across H&N and brain/CNS subgroups. OAR-dose subgroup analysis demonstrated a generally favorable direction of change among available structures, although direct structure-by-structure comparison was limited by differences in OAR availability between anatomical sites. Because the validation was retrospective and lacked full patient-level independence, the model should be interpreted as a robust institutional standardization tool rather than definitive evidence of external generalizability.

## Figures and Tables

**Table 1:** Characteristics of the final RapidPlan model, including the number of plans contained in the model, the structures considered, and the coefficients of determination (R^2^) obtained during training, describing the relationship between geometric features and dose.

	R^2^	Quantity	Outliers	Percentage (%)	Dosimetric	Geometric
PTV1	-	497	-	-	-	-
PTV2	-	252	-	-	-	-
PTV3	-	130	-	-	-	-
PTV4	-	26	-	-	-	-
Oral Cavity	0.54	227	69	30	0	69
Larynx	0.63	214	32	15	0	32
Spinal Cord	0.49	543	49	9	3	46
Salivary gland	0.73	733	46	6	5	43
Esophagus	0.78	27	15	56	0	15
Thyroid	0.81	255	10	4	1	9
Lips	0.81	125	4	3	0	4
Mandible	0.78	339	19	6	4	16
Optic Nerve	0.80	84	9	11	0	9
Optic Chiasm	0.79	215	4	2	0	4
Lens	0.72	301	16	5	1	15
Eye	0.68	276	7	3	0	7
Ear	0.84	207	13	6	1	13
Brain	0.85	204	19	9	3	18
Brainstem	0.73	300	8	3	3	7
Pituitary	0.71	29	4	14	0	4

**Table 2:** Dosimetric results. Values are reported as mean ± standard deviation. Delta is defined as RapidPlan minus clinical plan; negative values indicate a lower dose with RapidPlan for OARs. Confidence intervals refer to paired differences. For bilateral OARs, left and right structures were analyzed as paired structure instances when available.

Metric	*n*	Clinical Mean ± SD	RapidPlan Mean ± SD	Delta	95% CI	*p*-Value
PTV1 D98 (%Rx)	340	95.04 ± 5.60	94.95 ± 5.43	−0.09	−0.30 to 0.12	0.423
PTV1 D2 (%Rx)	340	102.89 ± 0.95	103.26 ± 1.08	0.37	0.25 to 0.48	<0.001
PTV1 SD (%Rx)	340	1.90 ± 1.68	1.98 ± 1.58	0.08	0.02 to 0.14	0.006
PTV2 D98 (%Rx)	268	75.33 ± 30.01	75.12 ± 29.93	−0.21	−0.43 to 0.00	0.055
PTV2 D2 (%Rx)	268	99.66 ± 9.37	100.05 ± 9.33	0.39	0.23 to 0.55	<0.001
PTV3 D98 (%Rx)	180	65.17 ± 31.43	64.88 ± 31.14	−0.29	−0.56 to −0.03	0.031
PTV4 D98 (%Rx)	47	63.20 ± 32.50	62.76 ± 32.29	−0.43	−0.79 to −0.07	0.019
Oral cavity Dmean (%Rx)	177	41.85 ± 16.99	34.23 ± 13.52	−7.62	−8.91 to −6.32	<0.001
Larynx Dmean (%Rx)	163	50.95 ± 22.40	43.38 ± 20.57	−7.57	−9.14 to −6.00	<0.001
Spinal cord Dmax (%Rx)	321	55.52 ± 15.23	55.42 ± 13.15	−0.10	−1.03 to 0.83	0.833
Brainstem Dmax (%Rx)	250	55.76 ± 27.06	53.62 ± 25.91	−2.14	−3.28 to −1.00	<0.001
Brain Dmax (%Rx)	165	74.34 ± 30.61	68.54 ± 32.34	−5.80	−7.12 to −4.48	<0.001
Thyroid Dmean (%Rx)	201	69.22 ± 26.50	63.93 ± 26.25	−5.29	−5.99 to −4.58	<0.001
Lips Dmean (%Rx)	104	27.99 ± 20.56	21.00 ± 17.48	−6.98	−8.47 to −5.50	<0.001
Mandible Dmax (%Rx)	273	96.09 ± 14.73	94.62 ± 15.75	−1.48	−2.01 to −0.95	<0.001
Optic chiasm Dmax (%Rx)	74	59.03 ± 32.61	56.82 ± 32.97	−2.21	−4.53 to 0.10	0.061
Pituitary Dmax (%Rx)	21	43.19 ± 37.44	35.15 ± 31.93	−8.04	−12.87 to −3.20	0.002
Salivary glands Dmean (%Rx)	587	34.42 ± 15.84	30.05 ± 14.68	−4.36	−4.80 to −3.93	<0.001
Optic nerve Dmax (%Rx)	181	35.62 ± 33.46	33.11 ± 32.23	−2.50	−3.71 to −1.30	<0.001
Lens Dmax (%Rx)	250	5.98 ± 5.44	6.26 ± 5.33	0.28	−0.01 to 0.57	0.060
Eye Dmax (%Rx)	227	24.68 ± 27.18	22.18 ± 23.71	−2.50	−3.59 to −1.41	<0.001
Ear Dmax (%Rx)	163	30.87 ± 31.07	24.75 ± 26.70	−6.13	−7.73 to −4.52	<0.001

**Table 3:** Training–validation overlap and cohort composition. Plan-level held-out cases were not included in the final model, whereas patient-level independence was unavailable.

Analysis Level	Training Set	Validation Set	Overlap	Training-Only	Validation-Only/Held-Out
Plan level	497	370	303 (81.9% of validation)	194	67 (18.1% of validation)
Unique-patient level	401	289	289 (100% of validation patients)	112	0

**Table 4:** Plan complexity analysis based on total monitor units (MUs). Negative Delta indicates lower MU with RapidPlan.

Cohort	*n*	Clinical MU Mean ± SD	RapidPlan MU Mean ± SD	Delta MU	95% CI	*p*-Value
All validation plans	370	764.2 ± 275.5	695.8 ± 210.3	−68.4	−89.4 to −47.4	<0.001
Plan-level overlap subset	303	769.0 ± 279.5	701.4 ± 220.3	−67.6	−91.0 to −44.2	<0.001
Plan-level held-out subset	67	742.5 ± 257.2	670.3 ± 156.1	−72.2	−121.1 to −23.3	0.004
H&N	267	792.1 ± 278.5	718.5 ± 201.2	−73.6	−96.2 to −51.1	<0.001
Brain/CNS	103	688.0 ± 259.5	636.2 ± 227.4	−51.8	−101.5 to −2.1	0.041
Wrocław	295	764.9 ± 288.2	699.2 ± 222.9	−65.7	−90.5 to −40.9	<0.001
Jelenia Góra	43	809.2 ± 224.6	698.3 ± 161.6	−110.9	−162.9 to −58.8	<0.001
Legnica	32	696.9 ± 199.3	660.8 ± 135.5	−36.1	−83.5 to 11.4	0.131

**Table 5:** Clinically relevant threshold achievement. Bilateral structures are reported per structure instance.

Structure/Metric	Threshold	*n*	Clinical Plans Meeting Threshold	RapidPlan Plans Meeting Threshold	Absolute Change
Oral cavity Dmean	<40 Gy	177	157/177 (88.7%)	174/177 (98.3%)	+9.6 pp
Larynx Dmean	<45 Gy	163	134/163 (82.2%)	150/163 (92.0%)	+9.8 pp
Non-parotid salivary glands Dmean (bilateral instances)	<26 Gy	618	465/618 (75.2%)	526/618 (85.1%)	+9.9 pp
Lens Dmax (bilateral instances)	<10 Gy	270	260/270 (96.3%)	254/270 (94.1%)	−2.2 pp
Eye Dmean (bilateral instances)	<38 Gy	242	229/242 (94.6%)	230/242 (95.0%)	+0.4 pp
Eye Dmax (bilateral instances)	<45 Gy	242	218/242 (90.1%)	222/242 (91.7%)	+1.7 pp

## Data Availability

The original contributions presented in this study are included in the article. Further inquiries can be directed to the corresponding authors.

## Abbreviations

The following abbreviations are used in this manuscript:

dA	Areal Differences
CD	Cook’s Distance
DVH	Dose–Volume Histogram
Dmax	Maximum Dose
Dmean	Mean Dose
GTV	Gross Tumor Volume
H&N	Head and Neck
ICD-10	International Classification of Diseases, 10th Revision
IMRT	Intensity-Modulated Radiation Therapy
KBP	Knowledge-Based Planning
KS	Kolmogorov–Smirnov
mZ	Modified Z-scores
OAR	Organ at Risk
PTV	Planning Target Volume
QA	Quality Assurance
R^2^	Coefficient of Determination
Rx	Prescribed Dose
SD	Standard Deviation
SIB	Simultaneous Integrated Boost
SR	Studentized Residuals
VMAT	Volumetric Modulated Arc Therapy
MCS	Modulation Complexity Score
MSCv	Modulation Complexity Score for VMAT
